# Supplementary material for: Synovial Fluid Derived from Human Knee Osteoarthritis Increases the Viability of Human Adipose-Derived Stem Cells through Upregulation of FOSL1
Source: Cells. 2023 Jan 15;12(2):330. doi: 10.3390/cells12020330 (PMC9856741; doi:10.3390/cells12020330)
Supplement: Supplementary file 1 [file cells-12-00330-s001.zip › cells-2071866-supplementary.pdf]

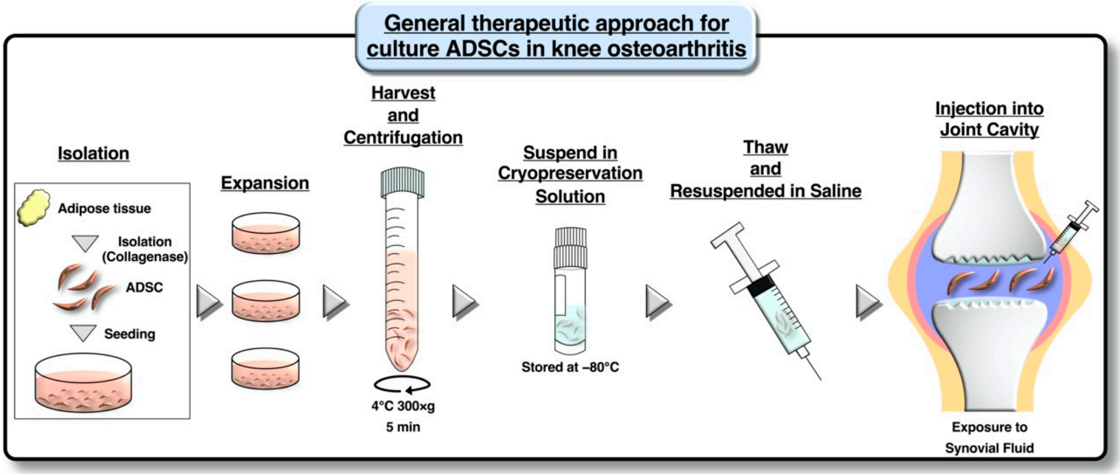

Figure S1. General therapeutic approach for culture ADSCs in Knee osteoarthritis.

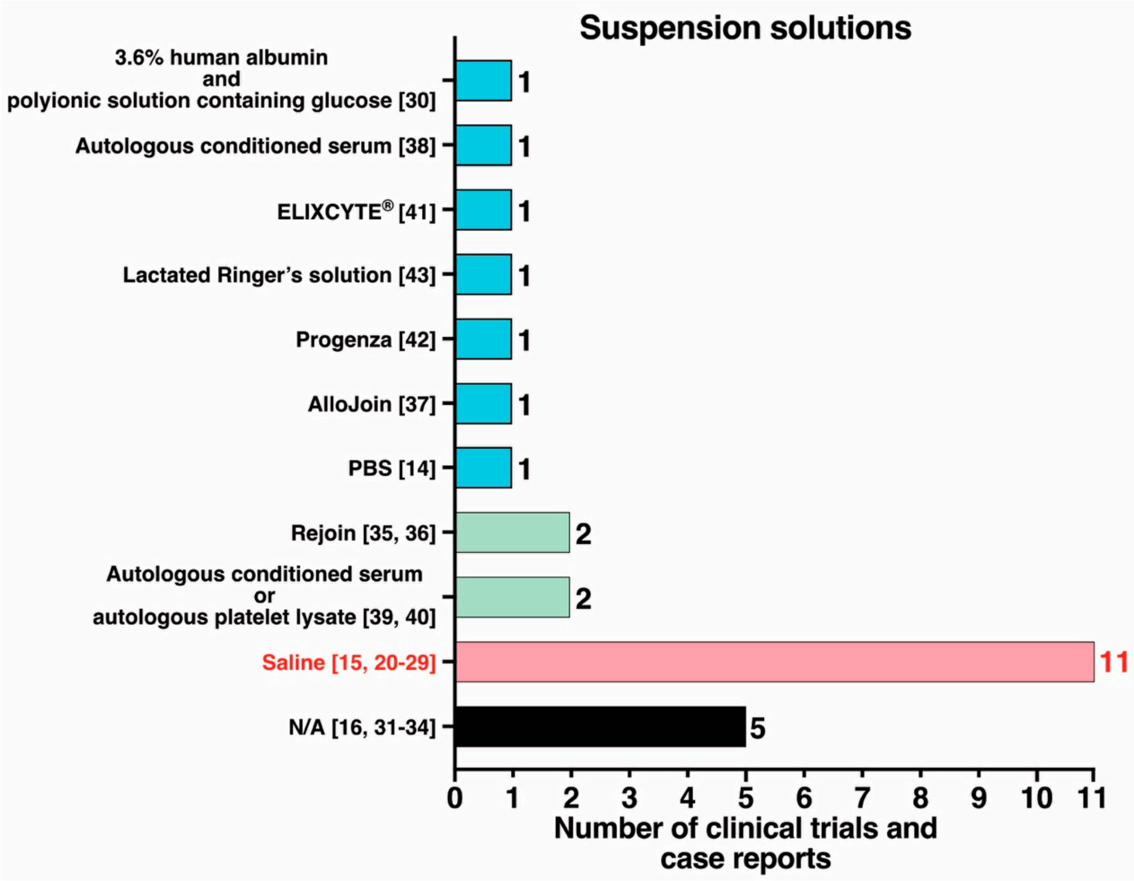

Figure S2. Summary of literature search with keywords related to cell therapy of Knee OA with culturing ADSCs. To explore the use of saline for ADSC cell suspension in Knee OA therapy, the clinical trial and case reports were searched in PubMed database. The keyword search was performed using the combination of "knee osteoarthritis" and "adipose derived stem cell". Of the 27 total hits, 11 had ADSCs suspended in saline.

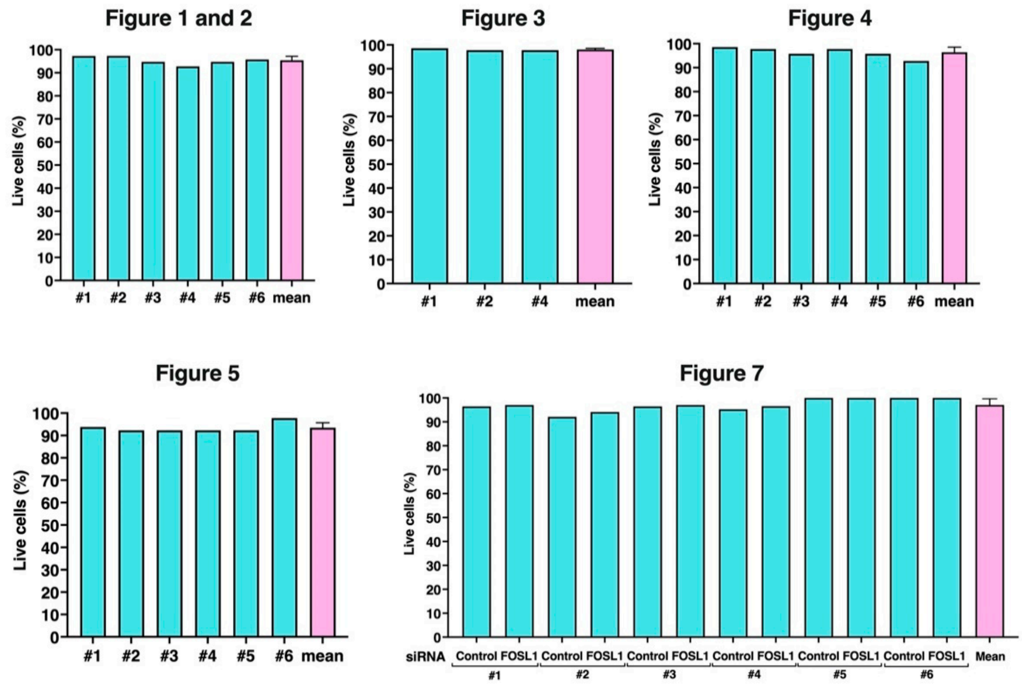

Figure S3. Quantification of cell viability by trypan blue staining of freeze-thawed ADSCs used in each experiment. Values are expressed as percentages of the number of viable cells relative to the total number of cells. The pink bar graph is expressed as mean ± S.D. of ADSCs suspended in each SF1-6.

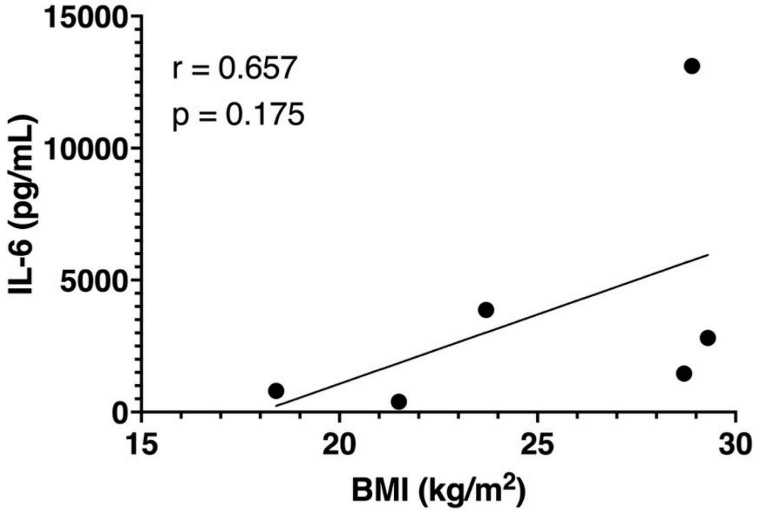

(**a**)

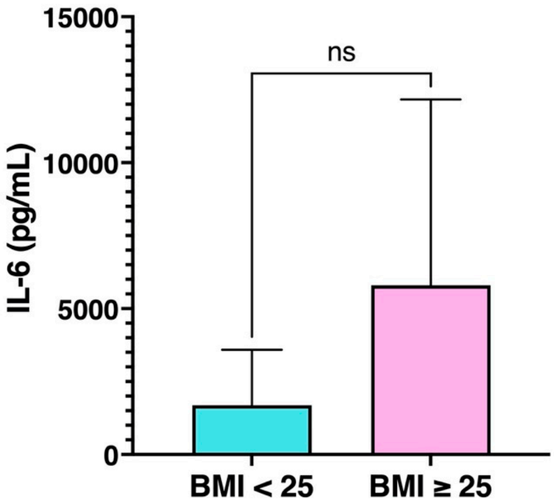

(**b**)

Figure S4. Correlation between BMI and IL-6 concentration in SF in knee OA patients. (**a**) Correlation between BMI and IL-6 concentration in SF in six patients; no correlation was observed between BMI and IL-6 concentration in SF (r = 0.657, p = 0.175). Spearman's rank correlation coefficient was used to test the correlation between BMI and IL-6 concentration. (**b**) Comparison of the IL-6 concentration in SF of low (< 25) and l high (≥ 25%) BMI patients. No significant difference was observed in the IL-6 concentration between high and low patients. Data are expressed as mean ± S.D.

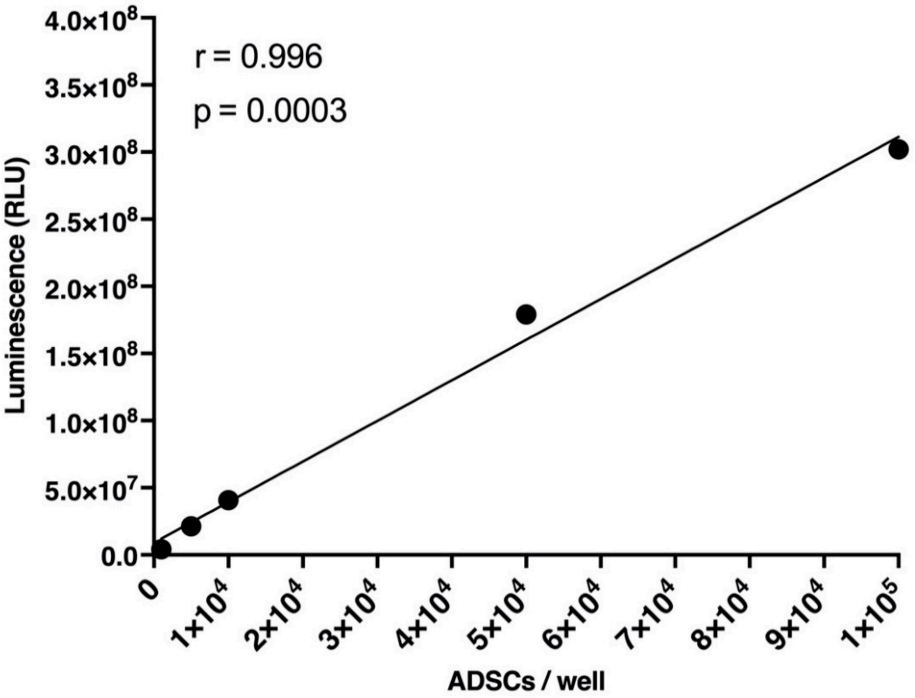
r = 0.996
p = 0.0003
Luminescence (RLU)
4.0×10^8
3.5×10^8
3.0×10^8
2.5×10^8
2.0×10^8
1.5×10^8
1.0×10^8
5.0×10^7
0.0
0
1×10^4
2×10^4
3×10^4
4×10^4
5×10^4
6×10^4
7×10^4
8×10^4
9×10^4
1×10^5
ADSCs / well

(**a**)

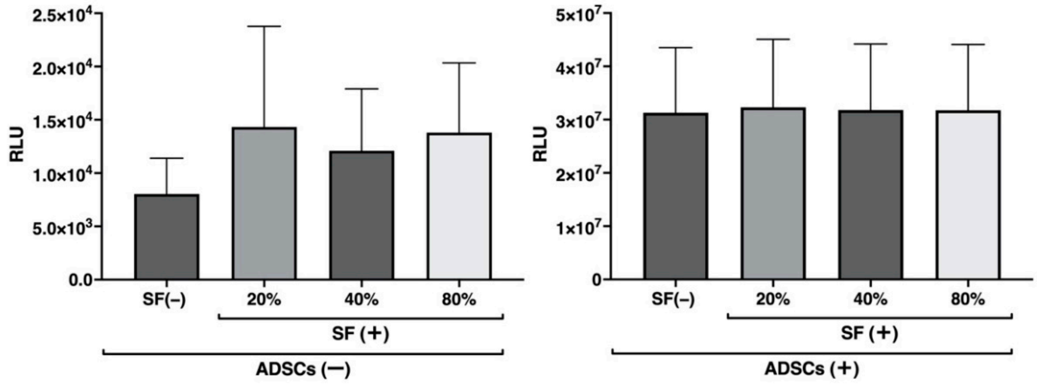
RLU
2.5×10^4
2.0×10^4
1.5×10^4
1.0×10^4
5.0×10^3
0.0
SF(–)
20%
40%
80%
SF (+)
ADSCs (−)
RLU
5×10^7
4×10^7
3×10^7
2×10^7
1×10^7
0
SF(–)
20%
40%
80%
SF (+)
ADSCs (+)

(**b**)

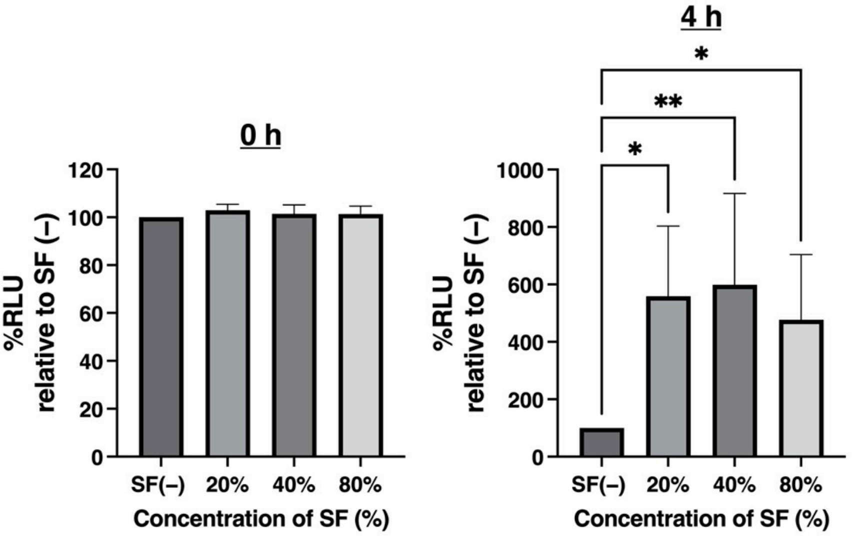
0 h
%RLU
relative to SF (–)
120
100
80
60
40
20
0
SF(–)
20%
40%
80%
Concentration of SF (%)
4 h
*
**
*
%RLU
relative to SF (–)
1000
800
600
400
200
0
SF(–)
20%
40%
80%
Concentration of SF (%)

(**c**)

Figure S5. Cell number correlates of ADSCs with luminescent output. (**a**) A linear direct relationship was found between luminescence measured using the CellTiter-Glo 3D cell viability assay and the number of cells in culture over five orders of magnitude. Serial 2- to 5-fold dilutions of ADSCs were performed in saline and plated in five formats. Cells were seeded at a density of 1×103 to 1×105 cells per well in 96-well plate and then an equal volume of Cell Titer-Glo Reagent was dispensed. Luminescence was recorded 30 min after the addition of CellTiter-Glo 3D Reagent. Spearman's rank correlation coefficient was used to test the correlation between Cell number and RLU. A linear relationship (r = 0.996) was observed between luminescent signal and cell number of ADSCs in each well. Spearman's rank correlation coefficient was used to test the correlation between BMI and IL-6 concentration. (**b**) ATP in SF at each concentration was measured by CellTiter-Glo 3D cell viability assay. The right panel showed that measured luminescence in the presence of the same cell number of ADSCs in Figure 2. The left panel showed that ATP in the SF at each concentration was measured by the CellTiter-Glo 3D cell viability assay and there was no difference in RLU values. (**c**) The percentage of RLU at 0 and 4 h for ADSCs suspended in each concentration of SF. The percentage of RLU in the samples at each concentration of SF was calculated by subtracting the RLU of each solution without ADSCs as a blank and dividing by the RLU at SF (-). Even when the SF contained ATPase, the percentage of viable cells was significantly increased 4 h after the addition of each concentration of SF compared with SF (-). These results indicated that each concentration of SF was shown not to interfere with the assay. Data are expressed as the mean ± S.D. * $p < 0.05$, and ** $p < 0.01$ are shown.

Table S1. Fold changes in genes with significantly differential expression in the DNA microarray analysis of ADSCs with SF obtained from each of the three patients.

Upregulated genes Log_2 [SF (+)/SF (-)]			
Gene	Patient No. #1	#2	#4
MIR221	0.503	0.659	0.615
NFATC2	0.586	0.537	0.481
SLC30A1	0.399	0.354	0.38
ABL2	0.27	0.295	0.308
KRTAP1-5	0.318	0.39	0.284
DKK1	0.377	0.245	0.289
DUSP14	0.331	0.258	0.216
NOCT	0.311	0.315	0.322
MIR222	0.387	0.293	0.437
USP38	0.273	0.252	0.325
ZSWIM6	0.189	0.29	0.253
SPRY2	0.346	0.221	0.205
MIR382	0.289	0.277	0.404
MIR22HG	0.306	0.261	0.296
FOSL1	0.183	0.225	0.25
HBEGF	0.375	0.295	0.245
ETS2	0.304	0.209	0.191
TJP2	0.252	0.269	0.226
BNC1	0.279	0.25	0.235
LINC00312|LMCD1	0.343	0.228	0.338
VGLL3	0.228	0.191	0.224
DUSP10	0.224	0.153	0.241
AMOTL2	0.276	0.216	0.22
ETS1	0.215	0.241	0.194
DAGLB|KDELR2	0.198	0.234	0.213
ZNF281	0.213	0.203	0.213
CTGF	0.226	0.103	0.101

Downregulated genes Log_2 [SF (+)/SF (-)]			
Gene	Patient No. #1	#2	#4
LOC101059936	-0.339	-0.111	-0.126
DUSP1	-0.129	-0.183	-0.14
CXCL2	-0.311	-0.17	-0.272
MGC24103	-0.3	-0.266	-0.317
UBC|RPS27A	-0.443	-0.193	-0.194
RNU11	-0.254	-0.236	-0.22
RGS2	-0.249	-0.347	-0.285
ARRDC3	-0.346	-0.295	-0.29
NFKBIA	-0.361	-0.246	-0.295
DDIT4	-0.341	-0.341	-0.319
ZFP36	-0.292	-0.278	-0.269
FOS	-0.397	-0.394	-0.334
SNORD14E	-0.604	-0.64	-0.617

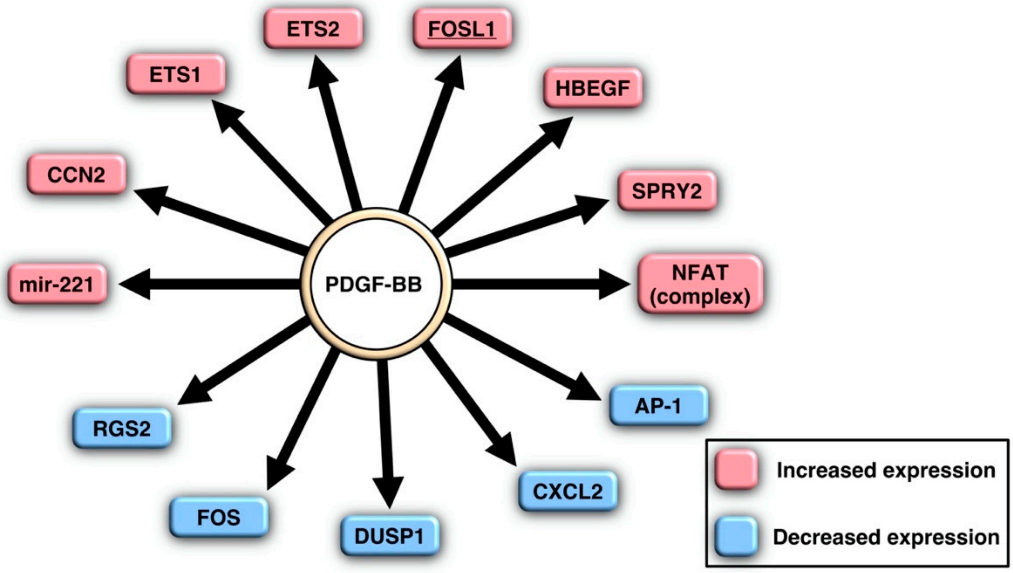

Figure S6. Ingenuity pathway analysis-identified gene network of PDGF-BB. IPA predicted the gene network associated PDGF-BB in 13 of 40 genes commonly altered by SF treated ADSCs.
